# Pathological Roles and Clinical Usefulness of Periostin in Type 2 Inflammation and Pulmonary Fibrosis

**DOI:** 10.3390/biom11081084

**Published:** 2021-07-22

**Authors:** Junya Ono, Masayuki Takai, Ayami Kamei, Yoshinori Azuma, Kenji Izuhara

**Affiliations:** 1 Shino-Test Corporation, 2-29-14 Oonodai Minami-ku, Sagamihara, Kanagawa 252-0331, Japan; masayuki.takai@shino-test.co.jp (M.T.); ayami.kamei@shino-test.co.jp (A.K.); yoshinori.azuma@shino-test.co.jp (Y.A.); 2 Division of Medical Biochemistry, Department of Biomolecular Science, Saga Medical School, 5-1-1 Nabeshima, Saga 849-8501, Japan; kizuhara@cc.saga-u.ac.jp

**Keywords:** periostin, type 2 inflammation, biomarker, IL-13, eosinophilic chronic rhinosinusitis, idiopathic pulmonary fibrosis

## Abstract

Periostin is known to be a useful biomarker for various diseases. In this article, we focus on allergic diseases and pulmonary fibrosis, for which we and others are now developing detection systems for periostin as a biomarker. Biomarker-based precision medicine in the management of type 2 inflammation and fibrotic diseases since heterogeneity is of utmost importance. Periostin expression is induced by type 2 cytokines (interleukin-4/-13) or transforming growth factor-β, and plays a vital role in the pathogenesis of allergic inflammation or interstitial lung disease, respectively, andits serum levels are correlated disease severity, prognosis and responsiveness to the treatment. We first summarise the importance of type 2 biomarker and then describe the pathological role of periostin in the development and progression of type 2 allergic inflammation and pulmonary fibrosis. In addition, then, we summarise the recent development of assay methods for periostin detection, and analyse the diseases in which periostin concentration is elevated in serum and local biological fluids and its usefulness as a biomarker. Furthermore, we describe recent findings of periostin as a biomarker in the use of biologics or anti-fibrotic therapy. Finally, we describe the factors that influence the change in periostin concentration under the healthy conditions.

## 1. Introduction

**Figure 1:**
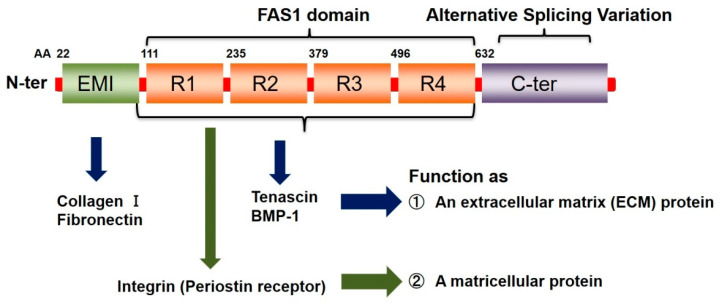
Schematic representation of periostin structural domains and function. FAS, Fasciclin; BMP, Bone morphogenetic protein.

Periostin is a 90kDa matricellular protein discovered in 1993 [1] and fully elucidated in 1999 [2] that plays a role in the pathogenesis of osteology, tissue repair, oncology, cardiovascular and respiratory diseases, and allergic inflammation [3]. The structure of periostin consists of one EMILIN-like (EMI) domain at the N-terminus, four repeating tandem of the fasciclin (FAS1)-1 domain in the middle, and an alternative splicing variant region at the C-terminus (Figure 1) [3,4]. The N-terminal region of periostin can interact with extracellular matrix (ECM) proteins (e.g., collagen Ι, fibronectin) and regulates cell-matrix organisation, resulting in remodelling and fibrosis [5,6,7]. Additionally, FAS1 domains bind to several integrins (e.g., αvβ1 [8,9], αvβ3 [9,10], αvβ5 [9,10], α6β4 [11], and αMβ2 [12] on the cell surfaces, causing activation of signalling pathways such as FAK [11,13], PI3-kinase [8,9,11,14], Akt [11,15], ERK [16], NF-κB [17,18] and STAT3 [16]. Because of these properties, periostin plays an essential role in regulating cell behaviour and ECM organisation. The unique feature of periostin can be attributed to the highly complex signalling pathways that lead to increased periostin production [3] (e.g., in response to stimulation by transforming growth factor-β (TGF-β), fibroblast growth factor (FGF), epidermal growth factor (EGF), bone morphogenetic protein-2 (BMP-2) or interleukin-4/-13 (IL-4/13)).

In 2006, Izuhara et al. [5] first identified that periostin is a downstream molecule of interleukin-4/-13 (IL-4/13), signature cytokines of type 2 inflammation, signalling, and first reported its relationship to allergic diseases, observing the accumulation of periostin in thickened basement membranes of bronchial epithelial cells in asthma patients. In 2009, Woodruff et al. [19] demonstrated that periostin expression in airway epithelial cells from subjects with asthma is a type 2 high inflammatory pattern in asthma, attracting attention as a potential surrogate marker for type 2 inflammation. In 2011, Corren et al. [20] reported that patients with high baseline serum periostin levels had more significant improvement in lung function with anti-IL-13 antibody (lebrikizumab) than did patients with low periostin levels, and the usefulness of periostin molecules as biomarkers or companion diagnostics of biologics for pulmonary and allergic diseases has been attractive. Although periostin is well-known as a useful biomarker for various diseases, many studies in the past decade have focused on allergic and fibrotic diseases, and reported periostin levels as a valuable biomarker, with its levels correlating with disease severity, prognosis and responsiveness to the treatment in atopic dermatitis (AD) [17,21,22], asthma [23,24,25,26] and idiopathic pulmonary fibrosis (IPF) [27,28,29,30]. Therefore, although periostin has been implicated in various pathogeneses, we will focus on allergic diseases and pulmonary fibrosis, for which we and others are now developing detection systems for periostin as a biomarker, in this article.

In this review, we first outline the clinical significance of previously reported type 2 inflammatory markers. Then, we summarise the pathological role of periostin in the development and progression of type 2 allergic inflammation and interstitial lung disease. Additionally, we describe recent developments in periostin biomarkers characteristics over the last decade.

## 2. Importance of Development of a Biomarker for Type2 Inflammation

**Figure 2:**
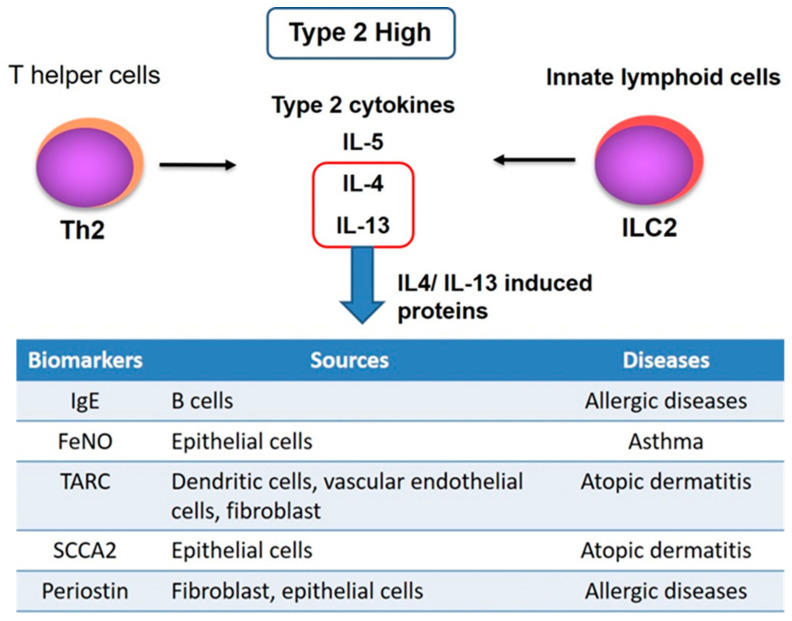
Type 2 biomarkers induced by IL-4/-13. Th, T helper; IL, interleukin; FeNO, Fractional exhaled nitric oxide; TARC, Thymus and activation-regulated chemokine; SCCA2, Squamous cell carcinoma antigens.

IL-13 is a type 2 cytokine that is thought to play a crucial role in allergic inflammation pathogenesis [31,32,33,34]. Overexpression of IL-13 in the lung in transgenic mice resulted in features typical of asthma, such as eosinophilic airway inflammation, increased mucus production, subepithelial fibrosis, and airway hyperresponsiveness [35,36]. Additionally, in mice sensitised with ovalbumin, neutralisation of IL-13 inhibited airway hyperresponsiveness, goblet cell metaplasia, and lung eosinophilia [37,38,39]. Previous clinical data have shown that atopic and non-atopic asthma patients have higher levels of IL-13 mRNA and IL-13 in sputum and bronchial biopsies than non-asthmatics [40,41]. Additionally, IL-13 serum levels serve as a biomarker of eosinophilic airway inflammation and are associated with asthma severity [42], but their levels in biological samples are very low and difficult to use clinically. Therefore, the development of surrogate biomarkers of type 2 cytokines is urgently needed, and various surrogate markers have been proposed as indicators of type 2 allergic inflammation (Figure 2). The following are some examples of these markers.

### 2.1. IgE

IgE is produced by IL-4-induced class switch of B cells to IgE-producing cells, and IgE plays a central role in airway inflammation in atopic asthma [43]. Serum IgE is generally higher in younger patients [44]. Even in severe asthma, young-onset atopic asthma has a different endophenotype from non-young-onset eosinophilic asthma, suggesting that IgE/allergic inflammation plays a significant role in asthma pathogenesis in younger patients. As an indicator for therapeutic management, serum IgE is considered a predictive marker of response to treatment with anti-IgE antibody [45].

### 2.2. Eosinophil

Eosinophils are produced through the IL-5-mediated pathway and may reflect eosinophilic inflammation in allergic diseases (ex. asthma, AD, and sinusitis) [46]. Sputum eosinophil percentage has been demonstrated to be a key marker in adjusting inhaled corticosteroid (ICS) dose, selecting patients for biologics use, predicting exacerbations, and improving asthma management [47,48]. The sputum eosinophil count is now considered the gold standard for understanding the pathogenesis of eosinophilic airway inflammation [49]. However, collecting sputum to assess eosinophilic inflammation is not practical in routine clinical practice because it is time-consuming and labour-intensive. Blood eosinophil counts are a useful biomarker that accurately reflects sputum eosinophil percentage [50]. Nowadays, the test of blood eosinophil counts is convenient and widely available on automated haematology machines. As an indicator for therapeutic management, peripheral blood eosinophil count is considered a predictive marker of response to treatment with anti-IL-5 antibody [50] and anti-IL-5 Rα antibody [51].

### 2.3. Fractional Exhaled Nitric Oxide (FeNO)

FeNO is produced in the airways by inducible NO synthase [52], an enzyme that IL-13 elevates [53,54]. FeNO levels are a surrogate marker for eosinophilic airway inflammation. High levels of FeNO have been associated with increased type 2 inflammation [55], increased risk of asthma exacerbations [56], and steroid insensitivity in asthmatics [57]. However, the correlation between FeNO and sputum eosinophils is indicated as modest level, especially under treatment with a high dose of ICS. FeNO has been investigated as a surrogate biomarker of eosinophilic inflammation in clinical trials of biologics for asthma treatment and has been shown to have properties that predict improved therapeutic efficacy [58]. As an indicator for therapeutic management, FeNO is considered a predictive marker of response to treatment with anti-IL-13 antibody [59] and anti-IL-4 Rα antibody [60].

### 2.4. Thymus and Activation-Regulated Chemokine (TARC)

TARC is a ligand (CCL17) for the chemokine receptor CCR4, expressed specifically to Th2 cells and is known as a marker of Th2-type immune responses [61,62]. TARC is produced by dendritic cells [63], keratinocytes [64] in the skin, vascular endothelial cells and fibroblasts [65] in the skin, airway epithelial cells [66] and platelets [67]. Serum TARC levels have been reported to increase with the severity of AD [62,66]. The problem with TARC levels is that the reference range differs between adults and children, with children showing higher levels [68], possibly due to an enhanced Th2-type immune response in the neonatal period [69].

### 2.5. Squamous Cell Carcinoma Antigens (SCCA2)

IL-22/-17, signature cytokines of type 17 inflammation and IL-4/-13 are associated with the pathogenesis of psoriasis and AD, respectively, and stimulation by these cytokines induce SCCA2 expression from airway epithelial cells and keratinocytes [31,70]. We previously showed that serum SCCA2 levels tightly correlated with clinical severity in patients with AD [71,72], and SCCA2 can be a biomarker for early diagnosis, estimation of clinical severity and type of disease, and assessing response to treatment. In the results of a multicenter analysis, SCCA2 and TARC serum levels were significantly higher in paediatric AD patients, with SCCA2 having better diagnostic accuracy [73]. Additionally, SCCA2 and TARC serum levels increased with clinical severity as estimated by Objective Scoring Atopic Dermatitis (O-SCORAD), but SCCA2 was better at distinguishing clinical severity in paediatric AD (AUC; SCCA2: 0.929, TARC: 0.871) [73]. Thus, SCCA2 was highly correlated with paediatric AD diagnosis (sensitivity 80%, specificity 95%) [73] and was approved as an in vitro diagnostics (IVD) for child AD in Japan in 2021.

## 3. Pathogenic Role of Periostin

Periostin has been indicated to be involved in the following broad pathological processes; inflammation, fibrosis, cell-to-cell or cell-to-matrix contacts, cell proliferation, and epithelial-mesenchymal transition (EMT) development [3,74]. Furthermore, we previously showed with immunostaining results that periostin is involved in the pathological role of many disease conditions, such as allergic inflammation [5,75], pulmonary [27], skin [17,76], hepatic [77] and ocular disease [78], and others also reported in kidney [79,80] and oral diseases [81], and tumour formation [74].

### 3.1. Discovery of Periostin

Periostin has been identified as a molecule expressed in the osteoblast cell line MCT3-E1 and was initially named osteoblast-specific factor 2 (OSF-2) [1]. Subsequently, it was found to be highly expressed in the periosteum and periodontal ligament and was called periostin [2]. Periostin is a member of the fasciclin family of proteins. Fasciclin family proteins have a FAS1 domain consisting of about 140 amino acids, a classic cell adhesion domain maintained in various plants and animals [82]. In addition to periostin, TGF-β-induced gene clone 3 (βig-h3), stabilin1, and stabilin2 belong to this family [82].

### 3.2. Pathological Role of Periostin in Allergic Inflammation and Interstitial Lung Disease

Periostin is a matricellular protein that regulates cell function and plays a pivotal role in the pathogenesis of allergic inflammation [17.32] and pulmonary fibrosis [83,84,85]. In airway inflammation, periostin in involved in both tissue remodelling and mucus production [3,86]. The mainstay of allergic inflammation such as bronchial asthma and AD is the type 2 inflammatory response [87,88], and thus periostin is speculated to have the pathological role of these states as downstream molecule of IL-13. In fact, periostin has been proven to be involved in the pathogenesis of almost all chronic allergic diseases, including asthma, eosinophilic sinusitis, AD, and allergic conjunctivitis [89].

**Figure 3:**
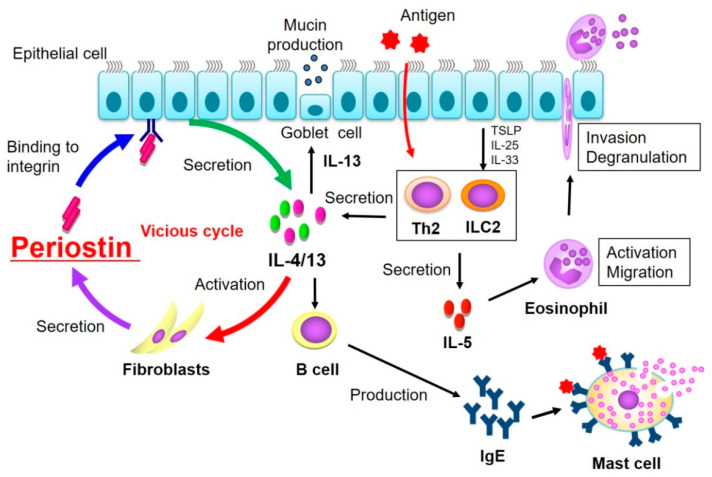
Summary of type 2 inflammatory response related to periostin in the pathogenesis of allergic diseases. TSLP, thymic stromal lymphopoietin; IL, interleukin; Th2, T helper 2 cells; ILC2, innate lymphoid cells.

In allergic inflammation response, periostin further amplifies the allergic inflammatory response (Figure 3). We previously reported that periostin activates NF-κB in keratinocyetes and concurrently activates NF-κB with other inflammation cytokines, such as TNFα or IL-1α in fibroblasts [90]. Furthemore, we found that periostin contributes to generating subepithelial fibrosis by constructing collagen fibrillogenesis by interacting with ECM such as collagen I, III and V, fibronectin, and tenascin-C in thickened basement membrane in asthmatic patients [5].

In the case of IPF, which is a typical fibrosis disease, myofibroblasts are considered to be the major pathogenic cells in IPF [91], as they produce abundant ECM and cause destruction of alveolar structures. In addition, the release of TGF-β, the most potent pro-fibrotic growth factor, has devastating effects, as TGF-β promotes apoptosis of epithelial cells and also induces prevention of apoptosis of lung fibroblasts [92]. In pulmonary fibrosis, it is reported that periostin is involved cross-talk with TGF-β, generating the expression of TGF-β downstream effector molecules involved in the pathogenesis of the disease [84].

## 4. Periostin as a Biomarker

Interestingly, Fingleton et al. [93] showed that serum periostin shows only a weak cross-correlation with other type 2 biomarkers; this suggests that periostin can complement the previously approved other markers and may be the only one characterised. The usefulness of periostin as a biomarker in the field of type 2 inflammation and fibrotic disease so far is reviewed below.

### 4.1. Periostin Assays

Periostin is present in very high concentrations in serum and can be easily measured by immunoassay. As far as we know, before 2011, serum periostin was measured by chemiluminescence ELISA using anti-periostin polyclonal antibodies, and most of the studies were in the fields of oncology, bone metastases and orthopaedics [94,95,96]. Since then, various monoclonal antibodies have been developed [29,97], and the study by Okamoto et al. in 2011 [27] was the first report of sandwich ELISA using monoclonal antibodies. Assay systems using monoclonal antibodies have the advantages of reduced non-specific reactions and high sensitivity. Since βig-h3 is a protein with high homology to periostin [82], assays using polyclonal antibodies may cross-react with these proteins. The assay system using monoclonal antibodies developed by us is about 20 times more sensitive than the assay system using polyclonal antibodies reported in the past [21,94]. This makes it possible to measure with high volume pre-dilution of the sample, and to reduce the interference of the matrix components of the sample on the measurement system. Additionally, the research results of Izuhara et al. [5] extended the focus of research to allergic inflammation. Various commercially ELISA kits and multiplex assays are available that can be used for research purposes. Additionally, automated reagents used in clinical trials have been developed to measure robust technical performance and precision. Roche Elecsys^®^ Periostin immunoassay was developed using an electrochemiluminescence assay (ECLIA) to provide an automated assay platform for measuring serum periostin with a quantification limit (LOQ) of 10 ng/mL [98].

Another automated assay, the ARCHITECT Periostin Immunoassay, based on the principle of chemiluminescent immunoassay (CLIA) method, was developed to measure the concentration of periostin in serum and has been used in asthma patients as an anti-IL-13 therapy [99]. It was used to evaluate the usefulness of periostin as a biomarker in two phase III studies (NCT02161757, NCT02194699) of tralokinumab. The LOQ for this method is 4 ng/mL, indicating a reliable and robust method for measuring serum periostin levels [99]. Additionally, its performance in several ELISA methods has been reported as follows.

ELISA method by Biomedica, using one monoclonal antibody recognising the R4 domain of periostin, and one polyclonal antibody recognising R1 and R2 regions, has a LOQ of 62.5 pmol/L, intra-assay CV: ≤3%, inter-assay CV: ≤6%, and proving that all isoforms can be detected [100]. The ELISA method by Shino-Test, using two monoclonal antibodies (SS18A and SS17B) recognising R1 and R4 domain of periostin, performs well with LOQ of 20 pmol/L, intra-assay and inter-assay CV: ≤3% [21,23]. This ELISA has been used in a wide range of research and clinical trial. Although there is limited information on the correlation between assays, it has been shown that there is a high correlation between Roche’s automated assay and Sino-Test’s ELISA (r = 0.9236) [101], as well as between R&D systems’ ELISA and Sino-Test’s ELISA (r = 0.9319) [25]. Conversely, there are differences in the measured values, higher levels with Shino-Test’s ELISA than others, among the assays. Thus, most of the currently available reagents are accurate, precise and show no significant interference in a range of values essential for routine clinical use. However, in contrast to other type 2 inflammatory biomarkers, periostin levels have still not been standardised, and caution is still needed in interpreting serum periostin concentrations obtained from studies using different assays (either electrochemiluminescence or ELISA). It has been mentioned that when using kits with poor reproducibility, the results should be judged carefully [102]. However, periostin is stable in serum, making it suitable for use in routine clinical practice.

### 4.2. Clinical Significance of Serum Periostin

Biomarkers for assessing patient status are required to serve as indicators for (1) screening, (2) diagnosis, (3) severity, (4) prognosis and (5) prediction of treatment response [87]. We summarise in Table 1 the allergic or fibrotic diseases with elevated serum periostin levels previously reported and the types of clinical significance as biomarkers. We have also shown that serum periostin increases with the severity of asthma and AD, indicating that it can be a severity biomarker. Additionally, we proved that periostin levels can be a marker for assessing the degree of skin fibrosis in scleroderma patients and a predictive marker of decreased respiratory function in IPF patients. Furthermore, it is well known that high serum periostin can be a marker for predicting the effect of biological therapy in asthma [86,103] or AD patients and a prognostic marker for assessing the risk of asthma exacerbation. In addition, periostin production is affected by several complications, including allergic rhinitis, chronic rhinosinusitis, AD, and aspirin intolerance. In a previous report that studied differences in periostin expression between the nasal cavity and upper airway, the nasal epithelium of the upper airway showed higher periostin expression than the lower bronchial epithelium [104]. In other words, the more peripheral the airway was, the lower the periostin expression was. Furthermore, in the neutrophilic asthma phenotype, periostin expression in the nasal cavity was lower than in healthy controls and in the eosinophilic phenotype. Thus, previous findings emphasise that nasal cells express higher periostin than bronchial cells. The serum periostin level also supports this phenomenon. Periostin has been recognised as a biomarker for chronic rhinosinusitis comorbid in patients with severe asthma; thus, we describe the following part regarding the utility of serum periostin for chronic rhinosinusitis (CRS). We then introduce the usefulness of periostin in IPF as a representative example of fibrotic disease.

In addition to allergic inflammation and fibrotic diseases, periostin has been reported to be involved in other pathogeneses. In particular, periostin is expressed in many tissues during foetal development, suggesting a role in development [130]. In periostin-deficient mice, collagen fibrils are poorly formed, and abnormalities in the formation of teeth and heart valve membranes are observed [131]. It has also been shown that periostin induces differentiation into heart valve progenitor cells and proliferation of cardiomyocytes during cardiac development and healing after myocardial infarction. Furthermore, it promotes tumor progression by inducing cell proliferation, tissue invasion and metastasis in various tumor cells [130]. The usefulness of serum periostin in non-allergic and non-fibrotic diseases is summarized in Supplemental Table S1, which is excluded from the subject of this article.

#### 4.2.1. Utility as a Biomarker for Eosinophilic Chronic Rhinosinusitis (ECRS)

There has been an increase in the number of patients with postoperative recurrent and refractory sinusitis, known as ECRS [104]. These cases are often associated with lower respiratory tract diseases such as bronchial asthma, and numerous eosinophilic infiltrates are found in the nasal sinus mucosa. Even after surgical treatment, recurrence often occurs, and treatment is complex. Appropriate management of the condition is essential to reduce the burden on patients and to reduce medical costs. Therefore, a severity classification that considers postoperative prognosis was developed, but the number of facilities that can implement this classification is limited because some of the diagnostic criteria include items that are not objective. Therefore, there is a need to establish an objective severity marker that can predict recurrence before surgery.

We analysed 369 surgical cases of CRS, and data showed that serum periostin levels were markedly elevated in severe ECRS [132]. Based on postoperative recurrence as the endpoint, the appropriate cut-off value of serum periostin was calculated to be 115.5 ng/mL, which showed a sensitivity of 60.7% and specificity of 61.9%. This diagnostic accuracy was not significantly different from the existing severity classification scores based on previously established in the JESREC study [133]. When the two groups were divided into high and low serum periostin groups using this cut-off value, there was a significant difference in the postoperative recurrence-free rate between the two groups. Based on these findings, we suggest that serum periostin may be a useful biomarker for evaluating disease severity in ECRS [104,132].

#### 4.2.2. Utility as a Biomarker for Idiopathic Pulmonary Fibrosis

Serum periostin has been reported to be a good biomarker for predicting decreased lung function, increased radiological fibrosis, and long-term prognosis [27,28,29,30]. It has been reported that the introduction of anti-fibrotic agents is effective in IPF treatment and that they have a certain inhibitory effect on fibrosis regardless of symptom severity [134]. In contrast, they are not recommended for all patients in terms of side effects [135,136]. Therefore, it is essential to properly evaluate the rate of progression of the disease when deciding on a treatment plan and providing guidance on daily life for patients with IPF. It is necessary to introduce active treatment at the earliest possible stage, especially for patients with rapid progression. However, there are currently no markers that can appropriately and assess the rate of progression early. Additionally, no uniform standard defines what constitutes a fast progression rate. However, it has been reported that a decrease in effortful lung capacity (FVC) or lung diffusing capacity (DLCO) for several months to one year is a prognostic factor of IPF [137]. Since IPF is an inferior prognostic type of idiopathic interstitial pneumonias (IIPs), the current practice is to differentiate IPF from IIPs, and imaging and pathological diagnosis are performed [138]. However, these are not objective or convenient and are not good indicators to evaluate the progression rate of each patient individually. Additionally, the serum markers (KL-6, SP-A, and SP-D), which are used as diagnostics for IIPs, cannot evaluate the type and rate of progression of IIPs. Furthermore, the rate of progression can be determined by assessing the degree of decline in respiratory function over a specific observation period, deterioration over time on HRCT images, and worsening of subjective respiratory symptoms. However, these methods require days to start treatment and are accompanied by worsening of the disease condition. Therefore, there is a need for a biomarker that can select patients with fast progression appropriately and early.

Periostin, which reflects a poor prognosis of respiratory function in idiopathic interstitial pneumonia [27,28,29,30], is expected to be helpful as a biomarker reflecting disease activity and severity in routine medical care. However, as mentioned earlier, serum levels have been reported to be elevated in various inflammatory diseases, and there is insufficient disease specificity. In other words, if the serum periostin level is high, it is expected to be a false positive due to the possibility of not being able to identify which fibrosis disease the subject is suffering from or due to a history of allergic disease. To solve this problem, by analysing the structure of periostin in vivo and using an assay system that recognises periostin monomers explicitly, we have developed a specific assay for IIPs with monomeric periostin detection, which is increased in diseases other than interstitial pneumonia [29]. The diagnostic performance of serum monomeric periostin in IPF patients using healthy controls was similar to existing markers such as KL-6 and SP-D. Furthermore, when we examined baseline serum concentrations and changes in respiratory function over the following six months, monomeric periostin showed the strongest correlation with changes in respiratory function [29]. We also analysed the relationship between low and high monomeric periostin levels and changes in respiratory function over six months and found that the amount of decrease in respiratory function was significantly greater in the high-level group. This trend was not observed for KL-6, SP-D, or LDH. Thus, monomeric periostin has high diagnostic ability. The fact that objective values are shown that are inversely correlated with changes in respiratory function in patients provides useful information to assist in diagnosis and prognosis evaluation for appropriate treatment. Furthermore, a recent study showed that monitoring changes in serum monomeric periostin is a prognostic prediction biomarker for patients with acute exacerbations in IPF patient [139].

Owing to the fact that interstitial lung disease (ILD) is a complex disease and, similar to IPF, it is a type of disease with poor prognosis, progressive fibrosing interstitial ILD (PF-ILD) has recently been proposed as a phenotype with progressive type of ILD that is unresponsive to conventional therapy [140]. Further studies are needed to determine whether periostin is a biomarker for PF-ILD.

### 4.3. Utility of Periostin in Local Biological Samples

Since periostin is easily secreted into biological fluids, periostin is detected in not only serum but also local biological samples such as tear [78], vitreous fluid [141,142,143], nasal lavage fluid [144], bronchoalveolar lavage fluid [145,146,147], sputum [148,149,150,151,152,153], exhaled breath condensate [154,155], saliva [156,157,158], gingival crevicular fluid [159] and urine [160,161,162,163] (Table 2). Therefore, periostin in several biological fluids may be used as a sensitive diagnostic or prognostic biomarker under various pathological conditions. We summarised the usefulness of periostin detection in local biological samples in Table 2. Salivary periostin has previously been studied in periodontal disease and is now being evaluated as an inflammatory research field. Salivary or sputum periostin concentrations were more sensitive than plasma/serum periostin in distinguishing between severe and non-severe asthma patients, suggesting that it is a useful marker for early diagnosis of asthma. In the next section, we discuss the usefulness of periostin using tears as a biological sample, which can be easily collected in a clinical setting.

#### Clinical Usefulness of Tear Periostin

Allergic conjunctival disease is an allergic inflammatory disease of the eye [164]. Various symptoms, mainly itching, redness, and oedema, cause a decrease in QOL and work efficiency in daily life, which is a problem [164]. It is essential to receive early and appropriate diagnosis and corresponding treatment to prevent the suffering caused by allergic conjunctival diseases. However, there are many similar conjunctival diseases, and definitive diagnosis is not easy. On the other hand, conventional blood-based allergy tests have disadvantages, such as not reflecting the local conditions of the eye. It is known that certain proteins that indicate eye conditions such as allergies and infections increase in tear fluid [165,166], suggesting the possibility of its application to diagnosis in the field of ophthalmology.

In patients with allergic conjunctival diseases, periostin is strongly expressed in conjunctival tissue due to type I allergic reaction of the conjunctiva, and high concentrations of periostin are found in tear fluid [78]. We measured the concentration of periostin in the tear fluid of 31 patients with allergic conjunctival diseases (atopic keratoconjunctivitis (AKC), seven patients with vernal catarrh (VKC), 17 patients with seasonal allergic conjunctivitis (SAC), 18 healthy subjects, 16 patients with AD without ocular allergic symptoms, and nine patients with allergic rhinitis, and tear periostin concentration increased significantly in patients with allergic conjunctival disease. When the cut-off value of tear periostin concentration was set at 6.0 ng/mL, the diagnostic accuracy of differentiation between the group of patients with allergic conjunctival disease and without ocular allergy was 85.7% in sensitivity and 88.0% in specificity [78]. Based on these findings, it was suggested that tear periostin could be a useful biomarker for diagnosing allergic conjunctival diseases.

### 4.4. Periostin as a Biomarker in Therapy

In recent years, several biomarkers have been proposed for personalized therapies. Biomarker-based precision medicine will provide significant clinical benefits in the selection of treatment responders, risk prediction, prognosis, and treatment design strategies in allergic diseases (asthma, CRS and AD) or IPF management. Asthma is a common disease that affects approximately 339 million people worldwide, up to 10% of whom are severely affected [167,168]. Patients with severe asthma have a reduced quality of life, frequent emergency room visits and hospitalisations for acute exacerbations, and consume most asthma-related healthcare resources [169]. These populations continue to have significant unmet clinical needs, some of which have been met by the recent development of biologics. To date, five biologics have been approved for patients with asthma, but unfortunately, not all of them are effective for all patients with severe asthma, and biomarkers are needed to identify those who are most likely to benefit from biologics. In terms of biomarkers, these include serum total IgE for anti-IgE therapy (omalizumab) and blood eosinophil counts for anti-IL-5 Rα (benralizumab), anti-IL-5 therapy (mepolizumab, reslizumab), and anti-IL-4Rα therapy (dupilumab), but they are not completely stratified [167]. The fact that the effective drugs vary from patient to patient may be attributed to the fact that in asthma, the underlying mechanism of airway inflammation that causes the disease is different in each patient, resulting in a diversity of diseases. There is an urgent need to identify and classify asthma phenotypes, predict clinical symptoms and treatment response, and discover new biomarkers to help identify new therapeutic targets.

When inhaled corticosteroids (ICS) are administered to asthma patients, periostin production is reduced, suggesting that airway inflammation is suppressed, and serum periostin levels have been demonstrated to decrease in response to systemic therapy and ICS therapy [170]. Conversely, if serum periostin remains elevated despite high dose inhaled corticosteroid therapy, there is a risk of airflow limitation due to steroid resistance. Thus, serum periostin levels can be used to assess treatment responsiveness.

In a clinical study, serum periostin has been evaluated to identify patients who are more effective with biologics and is at risk of developing asthma exacerbation and decreased lung function (Table 3). Interestingly, the use of some biologics and high doses of ICS has also been reported to decrease periostin levels [122,170,171,172,173]. Patients with severe asthma who were not specifically using Xolair had higher plasma periostin concentrations than healthy controls [156], and using Xolair was shown to reduce periostin levels in patients with severe asthma [170,173]. Thus, the use of Xolair treatment may affect the serum/plasma levels of periostin, suggesting periostin may be a possible monitoring marker. Tralokinumab, an anti-IL-13 monoclonal antibody, is being developed to treat severe uncontrolled asthma [167]. The results of the phase III clinical trial (STRATOS 1) showed that FeNO in the breath and serum periostin (SIDES algorithm: FeNO cut-off SIDES algorithm: FeNO cut-off value > 32.3 ppb: periostin cut-off value > 27.4 ng/mL) were the only biomarkers that could predict treatment response [167].

Like asthma, IPF has been defined as a disease with diverse patient prognoses. IPF is a devastating type of chronic fibrotic lung disease characterised by dyspnoea and progressive worsening of lung function. The median survival from diagnosis is 2.5 to 3.5 years, and the disease has a poor prognosis, but progression varies from patient to patient. Currently, only two anti-fibrotic agents (pirfenidone and nintedanib) are available to treat IPF in worldwide [176]. However, it is not possible to completely suppress the decline in respiratory function, and there is a clinical need for therapeutic intervention at an appropriate time for maximum therapeutic effect and decreased risk of side effects. Thus, IPF has heterogeneity in progression, suggesting that therapeutic management using biomarkers to predict clinical progression and treatment response is required. A study evaluating the therapeutic predictive value of pirfenidone found that the baseline values of each biomarker (CCL13, CCL18, COMP, CXCL13, CXCL14, periostin and YKL40) were prognostic [175], but periostin had different results in two different studies. We speculate that this different result was caused by differences in the characteristics of the patients in the entry population in the two trials. Since IPF is a heterogeneous population, it is difficult to normalize the stage and severity of the disease at entry, and the one-year prognosis is likely to be different in each trial. Therefore, further evaluation will be needed to conclude the efficacy of periostin as a treatment predictive biomarker of anti-fibrotic therapy for IPF.

In conclusion, periostin level may have utility as a biomarker of treatment response and disease progression in several severe pulmonary disorders and may be a valuable marker for recommending aggressive therapeutic intervention.

### 4.5. Variation of Serum Periostin in Healthy Subjects

As shown in Table 4, periostin levels in the healthy controls were slightly different in each study; this may be due to differences in antibodies reactivities used in the assay and differences in the reference materials. However, to understand the appropriate clinical utility of serum periostin levels, it is vital to understand better the factors that may influence these levels’ biological variation. For example, baseline periostin levels are high in paediatric serum [177], which may mask changes due to local release in the lesion site and reduce the usefulness of periostin biomarker. The gender difference for periostin expression was higher in males than in females in the bronchial epithelium of healthy controls [156]. Additionally, in the bronchial epithelium of asthma, only females expressed higher levels of POSTN mRNA (males did not) compared to healthy controls. This result implies that there is a gender difference in POSTN mRNA levels. However, previous studies have shown that the gender of the patient does not need to be considered when assessing serum periostin [178]. Concerning body composition, some studies have shown that BMI is negatively associated with serum periostin in healthy subjects and asthma patients [179,180], while others have shown that there is no significant correlation [181], and thus further discussion is needed. A study of daily variation in periostin levels in asthmatic and non-asthmatic adults showed that periostin levels tended to be higher in the morning than in the afternoon [182]. However, the range of variation was small, suggesting that the time of day of blood collection was unlikely to influence periostin evaluation and treatment decisions using periostin level.

## 5. Future Development as Periostin Biomarker

The role of type 2 biomarkers in identifying phenotypes of severe asthma has been extensively investigated, and the utility of combining biomarkers to identify exacerbation-prone subgroups has also been suggested. In the future, it will be necessary to select the best biomarkers, set cut-off values, and evaluate the best combinations of biomarkers for the selective use of various molecular target drugs.

Periostin is known to have post-translational modifications and multiple spliced isoforms have been reported to be present in varying proportions in different tissues and disease states [197]. However, there is still no isoform-differentiated biomarker evaluation study. In the future, the development of isoform-specific assays may contribute to the disease-specific diagnosis, and further development is expected.

## 6. Conclusions and Perspectives

This review discussed the current knowledge of periostin’s pathological role and utility as a biomarker with a focus on allergic and fibrotic diseases. Since periostin levels are altered by different clinical factors depending on the underlying pathological conditions in patients, periostin itself is not a disease-specific marker. However, periostin is a potential biomarker for targeting the phenotyping of several diseases. Furthermore, the new therapeutic strategies against type 2 inflammation or fibrotic disease may also be designed with periostin as a companion diagnostic. In conclusion, periostin levels in serum/plasma or other biological samples have been suggested as a biomarker for early diagnosis, evaluation of disease severity, prognosis prediction, and patient selection and a predictor of response to biological therapy in previous studies.

## Figures and Tables

**Table 1:** Summary of serum periostin expression in various allergic of fibrotic diseases and potential use as a clinical biomarker.

	Study Groups	Age	Type of Biomarker (BM)	Potential Use As Biomarker of Serum Periostin Levels
Ear	Eosinophilic otitis medeia	adult	diagnostic BM	Significantly higher in EOM patients than in COM patients [105].
Under/under airway	Asthma	children	diagnostic BM	Significantly higher in patients with athma than healthy control [106].
			severity BM	Significantly higher in children with severe asthma than with mild to moderate asthma [107].
		adult	severity BM	Significantly higher in the exacerbated asthma group than stable asthma and healthy control groups [108]. Correlated with airway wall thickness and sputum eosinophilia, and inversely correlated with airflow limitation [109]. High periostin level associated with a decline in FEV1 of 30 mL or greater per year [23].
			severity BM, stratification BM	Correlated with a specific phenotype of eosinophilic asthma, late-onset and often complicated by obstructive pulmonary dysfunction and nasal disorders [24]. Significantly higher in patients with positive DH than patients with negative DH [110]. Discriminated the eosinophilic asthma group from patients having eosinophilic or mixed granulocytic asthma [111]. Predicted AERD phenotype together with severe asthma phenotype [112]. Correlated with blood eosinophil counts and sputum eosinophil counts [112]. Significantly higher in comorbid AERD patients with more severe chronic rhinosinusitis than with less severe [112].
			monitoring BM	Significantly elevated in patients with steroid-naive asthma compared with the controls and patients with asthma who were treated with steroids [113].
			predictive BM	Patients with high pretreatment levels had greater improvement in lung function with lebrikizumab [20].
	Chronic rhinosinusitis with nasal polyps	adult	diagnostic BM	Significantly elevated in patients with CRS than controls [114]. Significantly higher in CRS patients with nasal polyps than with those without polyps [114].
			prognostic BM	Associated with the severity of CRSwNP, and postoperative recurrence of CRSwNP [107].
	Allergic rhinitis	children	differential diagnostic BM	Significantly higher in AR patients than AA patients [115].
		adult	monitoring BM	Associated with an effective response to SLIT [116]. Correlated with improvement the magnitude of RQLQ [116].
	Asthma with CRS	adult	predictive BM	Associated with the preventive effect of ESS for asthma exacerbations in CRS patients comorbid with asthma [117].
	Asthma-chronic obstructive pulmonary disease overlap	adult	differential diagnostic BM	Combined assessment of serum periostin and YKL-40 may identify ACO [118].
	Obstructive sleep apnea-hypopnea syndrome	adult	severity BM	Significantly increased in patients with OSAHS and positively correlated with the apnea-hypopnea index (AHI) and negatively correlated with the lowest saturation oxygen (LSaO_2_) and mean saturation oxygen (MSaO_2_) [119].
	Pulmonary tuberculosis	adult	differential diagnostic BM	Combination of MMP-1, MMP-9, and periostin data distinguished PTB from non-PTB patients [120].
	Allergic bronchopulmonary aspergillosis	adult	differential diagnostic BM	Significantly higher in ABPA patients than in SAFS patients [121].
Skin	Atopic dermatitis	children adult	diagnostic BM	Significantly higher in AD patients than in healthy controls [17,22].
		adult	diagnostic BM, severity BM	Significantly higher in patients with AD than in patients with healthy controls [21]. Positively correlated with disease severity [21].
			monitoring BM	Significant decreased during dupilumab treatment [122].
	Systemic sclerosis	adult	severity BM	Significantly elevated with dSSc compared with lSSc and control subjects [76]. MRSS was positively correlated with periostin levels in patients with SSc [76].
Lung	Idiopathic interstitial pneumonias	adult	diagnostic BM, prognosis BM	Significantly higher in IPF than healthy subjects and COP [27]. Inversely correlated with pulmonary functions [27,29].
			prognostic BM	Significantly correlated with the increase of honeycombing score on HRCT during a 6-month period [28]. Higher serum periostin level was a predictor of a shortened OS and TTE [28].
	Non-small cell lung cancer	adult	diagnostic BM	Significantly elevated in NSCLC patients than in healthy controls and/or BLD patients [95,123].
Liver	Non-alcoholic fatty liver disease	adult	diagnostic BM	Significantly higher in NAFLD than in control [124,125]. Among overweight and obese subjects, it was significantly higher in NAFLD subjects than those without NAFLD [124]. Associated with a higher risk for NAFLD among overweight and obese subjects [125].
	Hepatocellular carcinoma	adult	diagnostic BM	Significantly increased in HCC patients than healthy controls, patients with hepatolithiasis, and patients with liver cirrhosis [126]. Significantly associated with Edmondson grade [126].
	Cholangiocarcinoma	adult	differential diagnostic BM	significantly increased in patients with CCA than in healthy controls, patients with benign liver diseases and patients with breast cancer [127]. Significantly higher in patients with CCA compared to those patients with normal liver, liver cirrhosis, HCC and other malignancies [77].
Renal	Diabetic retinopathy	adult	diagnostic BM	Significantly associated with the presence of DR in patients with T2DM and is an independent risk factor of DR [128].
Esophagus	Eosinophilic esophagitis	adult	monitoring BM	Significant decrease after Proton-pump inhibitors [129].

EOM, Eosinophilic otitis media; COM, Chronic otitis media; FEV1, Forced Expiratory Volume in 1st second; DH, delayed hypersensitivity; AERD, aspirin-exacerbated respiratory disease; CRS, chronic rhinosinusitis; wNP, with nasal polyps; AR, Allergic rhinitis; AA, allergic asthma; asthma-chronic obstructive pulmonary disease overlap, ACO; YKL-40, Chitinase-3-like-1; OSAHS, obstructive sleep apnoea-hypopnea syndrome; PTB, Pulmonary tuberculosis; ABPA, Allergic bronchopulmonary aspergillosis; SAFS, Severe Asthma with Fungal Sensitivity; AD, Atopic dermatitis; SSc, Systemic sclerosis; dSSc, diffuse cutaneous systemic sclerosis; lSSc, Limited cutaneous systemic sclerosis; MRSS, the modified Rodnan total skin thickness score; IPF, Idiopathic interstitial pneumonias; HRCT, High resolution CT; OS, Overall survival; TTE, time-to-event; NSCLC, Non-small cell lung cancer; BLD, benign lung diseases; NAFLD, Non-alcoholic fatty liver disease; HCC, Hepatocellular carcinoma; CCA, Cholangiocarcinoma; DR, diabetic retinopathy.

**Table 2:** Summary of the presence of periostin in local biological samples and clinical utilities as a biomarker.

Specimens	Study Groups	Significant Differences (High Group vs. Low Group)
Tear	Ocular allergic diseases	AKC, VKC vs. SAC, healthy control [78].
Vitreous fluid	Diabetic retinopathy	PDR vs. non-diabetic ocular diseases [141,142].
		PDR, PVR vs. RRD, MH [143].
		PDR vs. MH, ERM [142].
Nasal lavage fluid	Chronic rhinosinusitis	CRSwNP vs. AR [144].
Bronchoalveolar Lavage Fluid	NSAIDs-tolerant asthma	N-ERD vs. NTA [145].
	Eosinophilic Pneumonia	AEP, CEP vs. healthy volunteers, sarcoidosis [146].
	Idiopathic eosinophilic pneumonia	EP vs. IPF, sarcoidosis [147].
Sputum	Asthma	asthma vs. healthy control [148].
		severe asthma vs. moderate to mild asthma [149].
		patients with persistent obstruction vs. without persistent obstruction [150].
		eosinophilic type vs. mixed granulocytic phenotype [151].
	ESS in CRS	with asthma vs. without asthma [152].
	Chronic rhinosinusitis comorbid asthma	CRS with asthma vs. CRS without asthma [153].
Exhaled breath condensate	Asthma	severe asthma vs. mild to moderate asthma [154].
	Asthma, COPD	asthma vs. COPD [155].
Saliva	Asthma	mild to moderate asthma vs. severe asthma [156].
	Chronic periodontitis	healthy control vs. chronic periodontitis [157].
	Chronic and aggressive periodontitis	non-periodontitis vs. chronic periodontitis [158].
Gingival crevicular fluid	Chronic periodontitis, gingivitis	gingivitis, healthy vs. chronic periodontitis [159].
Urine	Type 2 diabetic patients with nephropathy	DN vs. T2DM with normoalbuminuria [160].
	IgA nephropathy	IgA nephropathy vs. healthy control [161].
	Type 2 diabetes mellitus	microalbuminuria, macroalbuminuria vs. normoalbuminuria [162].
	Chronic allograft nephropathy	CAN vs. transplant and healthy controls [163].

AKC, Atopic keratoconjunctivitis; VKC, vernal keratoconjunctivitis; SAC, seasonal allergic conjunctivitis; PDR, Proliferative diabetic retinopathy; PVR, Proliferative vitreoretinopathy; RRD, Rhegmatogenous retinal detachment; MH, macular hole; ERM, idiopathic epiretinal membrane; CRSwNP, Chronic rhinosinusitis with nasal polyps; AR, allergic rhinitis; NSAIDs, Non-Steroidal Anti-Inflammatory Drugs; N-ERD, NSAID-exacerbated respiratory disease; NTA, NSAID-tolerant asthmatic; AEP, Acute eosinophilic pneumonia; CEP, chronic eosinophilic pneumonia; EP, Eosinophilic pneumonia; IPF, Idiopathic pulmonary fibrosis; ESS, Endoscopic sinus surgery; CRS, Chronic Rhinosinusitis; COPD, Chronic obstructive pulmonary disease; DN, Diabetic nephropathy; T2DM, type 2 diabetes mellitus; CAN, Chronic allograft nephropathy.

**Table 3:** Summary of clinical utilities as a predictive biomarker of treatment.

Drug	Manufacture	Target	Diseases	Specimens	Outcomes of Periostin As Predictive or Monitoring Biomarker
Lebrikizumab	Genentech	IL-13	Asthma	Serum	Patients with high pretreatment levels of serum periostin had greater improvement in lung function with lebrikizumab than did patients with low periostin levels [20].
				Serum	Lebrikizumab-treated subjects with elevated baseline levels of peripheral blood eosinophils, serum IgE, or periostin exhibited a greater reduction in late asthmatic response [171].
				Serum	Treatment with lebrikizumab reduced the rate of asthma exacerbations, which was more pronounced in the periostin-high patients (60% reduction) than in the periostin-low patients (5% reduction) [59].
				Serum	Lung function also improved the following: lebrikizumab treatment, with greatest increase in FEV1 in periostin-high patients [58].
Omalizumab	Genentech	IgE	Asthma	Serum	There was a large difference in reduction in exacerbations after 48 weeks with omalizumab treatment between the high and low periostin groups (30% vs. 3%) [172].
				Serum	Omalizumab significantly reduced serum periostin levels at 4 and 8 weeks after the start of the treatment [170].
				Serum	Serum periostin levels decreased in omalizumab-treated patients in comparison to conventionally treated patients. This effect was remarkably apparent only if CRSwNP was not present [173].
Mepolizumab	GlaxoSmithKline	IL-5	Asthma	Nasal secretion	Nasal periostin levels decreased significantly after 8 weeks of treatment with mepolizumab [172].
Dupilumab	Regeneron/Sanofi	IL-4 Rα	Asthma	Serum	Peripheral blood eosinophil and FeNO concentrations were associated with lower exacerbation frequency and improved respiratory function, whereas periostin concentrations were associated only with improved respiratory function [60].
			AD	Serum	During dupilumab treatment, disease severity-related serum periostin significantly decreased [122].
Tralokinumab	AstraZeneca	IL-13	Asthma	Serum	FeNO (>32.3 ppb) and periostin (>27.4 ng/mL) were identified as the only biomarkers potentially predictive of treatment effect [167].
			IPF	Serum	Tralokinumab did not demonstrate a significant change in percent predicted FVC from baseline to Week 52 for either the periostin-high or the periostin-low group [174].
Pirfenidone	Genentech	TGF-β1, TNF-α	IPF	Serum	Several baseline biomarkers (CCL13, CCL18, CXCL13, CXCL14, periostin, and YKL40) were prognostic for progression outcomes with pirfenidon treatment [175].

AD, Atopic dermatitis; IPF, Idiopathic pulmonary fibrosis; IL, interleukin; Rα, Receptor alpha; TGF-β, Transforming growth factor-beta; TNF-α, Tumour Necrosis Factor-α; FeNO, Fractional exhaled nitric oxide; FEV1, Forced Expiratory Volume in one second; CCL, C-C motif chemokine; CXCL, C-X-C motif chemokine; YKL-40, Anti-Chitinase-3-like protein 1.

**Table 4:** Summary of serum periostin levels of healthy controls between each kit reported previously.

Kit Manufacture	Method	Age	Subject Number	Periostin (ng/mL)
Shino-Test Corp.	ELISA	Children	30	74.0 ^a)^ [69.75–80.0] ^e)^ [183]
			23	71.0 ^a)^ [57.5–80.0] ^e)^ [184]
		Adult	113	89.7 ^b)^ ± 21.2 ^c)^ [185]
			230	66.1 ^a)^ (0.14) ^e)^ [179]
			11	57.0 ^a)^ [39.0–63.0] ^e)^ [24]
			25	56 ^a)^ [49–63] ^e)^ [21]
			66	39.1 ^b)^ ± 3.0 ^d)^ [27]
R&D Systems	ELISA	Children	20	93.4^ a)^± 22.3 ^c)^ [115]
			60	32.7 ^b)^ ± 2.6 ^d)^ [186]
		Adult	110	37.4 ^a)^ [32.9–48.4] ^e)^ [187]
			17	24.6 ^b)^ ± 11.3 ^c)^ [188]
Biomedica	ELISA	Adult	45	966.9 ^b)^ ± 195.4 ^c)^ pmol/L [189]
			128	958 pmol/L ^b)^ [190]
			24	864 ^a)^ ± 269 ^c)^ pmol/L [100]
Genentech, Inc.,	ELISA	Adult	44	NS [191]
Roche Diagnostics	ECLIA		480	51.2 ^a)^ (11.9) ^e)^ [192]
Abbott Laboratories	CLIA	Adult	757	16 ^b)^ (5–56) ^f)^ [99]
Thermo Fisher Scientific	ELISA	Adult	10	3.72 ^b)^ ± 0.33 ^d)^ [193]
			10	40.0 ^b)^ [30.3–45.0] ^e)^ [194]
Cloud-Clone-Corp.	ELISA	Adult	24	1021 ^a)^ [807–1941] ^e)^ [195]
Bioassay Technology	ELISA	Children	31	23.6 ^b)^ ± 7.3 ^c)^ [22]
eBioscience	ELISA	Adult	160	21.27 ^b)^ ± 3.42 ^c)^ [123]
USCN Life Science Inc.	ELISA	Adult	40	299.29 ^b)^ ± 42.32 ^c)^ [196]

^a)^ median; ^b)^ mean; ^c)^ standard deviation (SD); ^d)^ standard error (SE); ^e)^ interquartile ranges; ^f)^ range; NS, Data not shown

## Supplementary Materials

The following are available online at https://www.mdpi.com/article/10.3390/biom11081084/s1, Table S1: Summary of clinical utilities of serum periostin levels in non-allergic and non-fibrotic diseases.
