# Supplementary material for: Pathological Roles and Clinical Usefulness of Periostin in Type 2 Inflammation and Pulmonary Fibrosis
Source: Biomolecules. 2021 Jul 22;11(8):1084. doi: 10.3390/biom11081084 (PMC8391913; doi:10.3390/biom11081084)
Supplement: Supplementary file 1 [file biomolecules-11-01084-s001.zip › biomolecules-1285300-supplementary.pdf]

Supplemental Table S1. Summary of clinical utilities of serum periostin levels in non-allergic and non-fibrotic diseases.

	Study Groups	Age	Type of biomarker (BM)	Potential use as biomarker of serum periostin levels
Head/brain	Stroke	adult	severity BM	・Significantly increased on day 6 after stroke, and positively correlated with the NIHSS scores and stroke volume. [S1]
	Aneurysmal subarachnoid hemorrhage	adult	prognostic BM	・Significantly higher in patients with poor outcome at 12 months than patients with good outcome. [S2]
	Traumatic brain injury	adult	diagnostic BM	・Significantly increased in the TBI patients. [S3]
	Intracerebral hemorrhage	adult	severity BM, prognostic BM	・Significantly higher in ICH patients, and its level were positively related to NIHSS score and hematoma volume. [S4]
Nervous system	Spinal cord injury	adult	diagnostic BM	・Significantly higher in SCI patients than in controls. [S5]
Connective tissue	Kawasaki disease	children	diagnostic BM	・Significantly elevated in Kawasaki disease patients compared with febrile controls. [S6]
Bone	Fibrous dysplasia of bone	adult	diagnostic BM	・Significantly higher in patients with FD compared to controls, especially in those with a history of fracture, polyostotic forms or McCune-Albright syndrome. [S7]
Oral cavity	Oral lichen planus	adult	diagnostic BM	・Significantly higher in OLP group than control group. [S8]

Obstetrics and gynecology	Spontaneous pregnancy loss	adult	predictive BM	・Significantly reduced in patients with spontaneous pregnancy loss, and can be used as a predictive marker for the success of endometrial implantation. [S9]
	Polycystic ovary syndrome	adult	diagnostic BM	・Significantly elevated in PCOS women compared with controls. [S10]
	Endometrial cancer	adult	diagnostic BM	・Significantly higher in women with endometrial cancer than in those without cancer. [S11]
Infection	Acute rheumatic fever	children	diagnostic BM	・Significantly higher in patients with ARF on admission than in the control group<br>・Significantly decreased in the patient group after treatment. [S12]

National Institutes of Health Stroke Scale, NIHS; TBI, Traumatic brain injury, ICH, Intracerebral haemorrhage; SCI, Spinal cord injury; FD, Fibrous dysplasia of bone; OLP, Oral lichen planus; PCOS, Polycystic ovary syndrome; ARF, acute rheumatic fever

Supplemental References

[S1] X. He, Y. Bao, Y. Shen, E. Wang, W. Hong, S. Ke, X. Jin. Longitudinal evaluation of serum periostin levels in patients after large-artery atherosclerotic stroke: A prospective observational study. Sci Rep. 2018; 8(1): 11729.

[S2] W. Luo, H. Wang, J. Hu. Increased concentration of serum periostin is associated with poor outcome of patients with aneurysmal subarachnoid hemorrhage. J Clin Lab Anal. 2018; 32(5). e22389.

[S3] X. Q. Dong, W. H. Yu, Q. Du, H. Wang, Q. Zhu, D. B. Yang, Z. H. Che, Y. F. Shen, L. Jiang. Serum periostin concentrations and outcomes after severe traumatic brain injury. Clin Chim Acta. 2017; 471: 298-303.

[S4] W. J. Ji, X. M. Chou, G. Q. Wu, Y. F. Shen, X. G. Yang, Z. F. Wang, L. X. Lan, X. G. Shi.

Association between serum periostin concentrations and outcome after acute spontaneous intracerebral hemorrhage. Clin Chim Acta. 2017; 474: 23-27.
[S5] L. Maïmoun, F. Ben Bouallègue, A. Gelis, S. Aouinti, T. Mura, P. Philibert, J. C. Souberbielle, M. Piketty, P. Garnero, D. Mariano-Goulart, C. Fattal. Periostin and sclerostin levels in individuals with spinal cord injury and their relationship with bone mass, bone turnover, fracture and osteoporosis status. Bone. 2019; 127: 612-619.
[S6] R. Reindel, K. Y. Kim, S. C. Baker, S. T. Shulman, E. J. Perlman, M. W. Lingen, C. Trevenen, A. H. Rowley. Periostin is upregulated in coronary arteriopathy in Kawasaki disease and is a potential diagnostic biomarker. Pediatr Infect Dis J. 2014; 33(6): 659-661.
[S7] H. Guerin Lemaire, B. Merle, O. Borel, D. Gensburger, R. Chapurlat. Serum periostin levels and severity of fibrous dysplasia of bone. Bone. 2019; 121: 68-71.
[S8] Z. R. Zhang, L. Y. Chen, H. Y. Qi, S. H. Sun. Expression and clinical significance of periostin in oral lichen planus. Exp Ther Med. 2018; 15(6): 5141-5147.
[S9] S. Eroglu, E. Colak, O. H. Erinanc, D. Ozdemir, M. U. Ceran, U. Tasdemir, S. Kulaksizoglu, E. E. Ozcimen. Serum and placental periostin levels in women with early pregnancy loss. J Reprod Immunol, 2020; 140: 103138.
[S10] X. Chen, L. Huo, L. Ren, Y. Li, Y. Sun, Y. Li, P. Zhang, S. Chen, G. Y. Song. Polycystic Ovary Syndrome is Associated with Elevated Periostin Levels. Exp Clin Endocrinol Diabetes. 2019; 127(9): 571-577.
[S11] S. Unuvar, R. Melekoglu, N. B. Turkmen, E. Yilmaz, S. Yasar, H. Yuce. Comparison of preoperative serum neopterin, periostin, indoleamine 2,3-dioxygenase, YKL-40, and tenascin-C levels with current tumor markers for early-stage endometrial cancer. Int J Gynaecol Obstet. 2021; doi: 10.1002/ijgo.13666.
[S12] S. Epçaçan, E. Yücel. Serum periostin levels in acute rheumatic fever: is it useful as a new biomarker?. Paediatr Int Child Health. 2020; 40(2): 111-116.
